# Isolation of Monoclonal Neutralizing Single-Domain Antibodies Against Clostridioides difficile Toxin B

**DOI:** 10.32607/actanaturae.27874

**Published:** 2026

**Authors:** I. A. Alekseeva, A. S. Ungur, I. A. Favorskaya, A. I. Tukhvatulin, D. V. Shcheblyakov, A. I. Korobkova, M. E. Komyakova, O. L. Voronina, N. N. Ryzhova, E. I. Ermolova, M. S. Kunda, D. Yu. Logunov, M. M. Bobrova, V. V. Makarov, S. M. Yudin, A. L. Gintsburg

**Affiliations:** Gamaleya National Research Center of Epidemiology and Microbiology of the Ministry of Health of the Russian Federation, Moscow, 123098 Russia; Centre for Strategic Planning and Management of Biomedical Health Risks of the Federal Medical and Biological Agency, Moscow, 119121 Russia

**Keywords:** Clostridioides difficile, CDI, toxin B, TcdB, single-domain antibodies, VHH

## Abstract

The Clostridioides difficile infection (CDI) is one of the most common nosocomial infections around. The key pathogenicity factors of this bacterium include the toxins A and B that cause the disease symptoms. Neutralization of these toxins is one of the promising strategies for CDI treatment. Here, we isolated a panel of single-domain antibodies to the toxin B CROPs domain. Two antibodies, TB5A7 and TB4A8, exhibiting potent neutralizing activity were modified to produce homodimeric forms. The TB5A7 and TB4A8 dimers were characterized by enhanced neutralizing activity and protected animals from a toxin B lethal challenge. The obtained antibodies may be used to develop new agents for CDI treatment.

## INTRODUCTION

Clostridioides difficile is a Gram-positive anaerobic bacterium that is responsible for the development of the Clostridioides difficile infection (CDI). CDI is the leading cause of antibiotic-associated diarrhea, with clinical symptoms ranging from mild to life-threatening conditions such as toxic megacolon and intestinal perforation. CDI is characterized by significant morbidity and mortality worldwide [1, 2].

The pathogenesis of the disease is primarily associated with two bacterial exotoxins, toxin A (TcdA) and toxin B (TcdB), that disrupt the function of intestinal epithelial cells, causing diarrhea and colitis [3]. TcdA and TcdB belong to the family of large clostridial toxins that are glycosyltransferases inactivating Rho family GTPases. Inactivation of GTPases results in damage to the cytoskeleton structure and destruction of tight junctions, resulting in increased permeability of the intestinal epithelium and inflammation [4, 5]. TcdA and TcdB contain several functional domains, including a glycosyltransferase domain (GTD), an autoprotease domain (APD), a delivery domain (DD), and a combined repetitive oligopeptides (CROPs) domain. The mechanism of the toxin’s action involves several stages: CROPs- and DD-mediated binding to receptors on the target cell surface and endocytosis; conformational changes of the toxin molecule in an acidic endosomal environment; pore formation and translocation of the GTD and APD domains into the cytoplasm; and autoprocessing and GTD release, followed by GTPase glycosylation [6]. In addition to toxins A and B, some C. difficile strains produce a third toxin, known as a binary toxin or C. difficile transferase (CDT). This toxin functions as an actin-specific ADP-ribosyltransferase that causes actin depolymerization [6].

The relative importance of TcdA and TcdB to the infection pathogenesis has not been completely established yet. Initially, TcdA was thought to be the key virulence factor [7, 8]. But later, pathogenic C. difficile strains that produced toxin B alone were isolated from patients [9]. Further studies established the key role of TcdB in the development of severe infection symptoms [10].

Standard CDI treatment includes the use of antibacterial drugs [2]. However, due to the high rate of recurrent infection and the emergence of resistant strains, there is a need to develop alternative therapeutic strategies. Because disease symptoms are mainly associated with the production of the bacterial toxins A and B, the development of agents that neutralize their effects is of particular interest. One of the promising approaches to producing these agents is the use of technologies for monoclonal antibody identification [11,12, 13, 14].

To obtain neutralizing antibodies that inhibit TcdB binding to receptors on the target cell surface, we isolated a panel of monoclonal single-domain antibodies to the TcdB CROPs domain. Two clones, TB5A7 and TB4A8, possessing the most pronounced neutralizing activity were isolated from the panel. Dimerization of the selected clones improved their neutralizing properties. Analysis of protective activity in vivo revealed that the TB5A7 and TB4A8 dimers were able to protect animals from a lethal TcdB challenge.

## EXPERIMENTAL

**Alpaca immunization**

An alpaca (Vicugna pacos) was immunized with five consecutive administrations of the recombinant TcdB CROPs domain (100 μg subcutaneously) at 10- to 14-day intervals. Freund’s complete adjuvant (Sigma, USA) was used for the first immunization, and Freund’s incomplete adjuvant was used for consecutive administrations. Five days after the last administration, a 50 mL blood sample was collected for mononuclear cell isolation. The animal study was approved by the Biomedical Ethics Committee of the Gamaleya National Research Center of Epidemiology and Microbiology of the Russian Ministry of Health and was conducted at the “Russian Alpacas” farm (Pokhodkino village, Moscow Region) under a research agreement.

**Single-domain antibody library generation**

A single-domain antibody library was generated as previously described [14]. Total RNA was isolated from peripheral blood mononuclear cells using the Trizol reagent (Thermo Fisher Scientific, USA). Then, the RNA was reverse transcribed using a SuperScript™ IV kit (Thermo Fisher Scientific). The resulting cDNA was used to amplify the regions encoding the VHH domains of heavy-chain antibodies (a special category of antibody consisting of a homodimer of truncated heavy chains). The VHH sequences were cloned into the pHEN phagemid vector [15]. The Myc-tag and 6xHis-tag were introduced into the antibody sequences during cloning. The resulting recombinant phagemid vectors were used to transform electrocompetent E. coli strain TG1 cells. The following day, the number of transformed cell colonies (size of the generated library) was counted. In addition, 40 colonies were selected for library quality control: the percentage of colonies carrying the vector with the right insert size was assessed. Analysis was performed using PCR; the percentage of positive colonies exceeded 90%.

**Phage library preparation and biopanning**

The phage library was prepared according to the previously described method [14]. For biopanning, the TcdB CROPs domain was immobilized onto the wells (1 μg per well) of an immunoassay plate (Nunc MaxiSorp, Thermo Fisher Scientific). After immobilization, the wells were washed with phosphate-buffered saline (PBS) containing 0.1% Tween 20 (PBST) and blocked using a blocking buffer (5% dry skimmed milk in PBST). Next, the wells were complemented with 100 µL of a blocking buffer containing ~1011 recombinant phage particles and incubated at 37°C for 1 h. Phage particles that did not bind to the antigen were removed by washing the wells with PBST. Antigen-bound phage particles were eluted with a trypsin solution (1 mg/mL). The eluted phages were used to infect TG1 cells, yielding an antibody library enriched in antigen-binding clones. Two rounds of biopanning were performed.

**Enzyme-linked immunosorbent assay (ELISA)**

For monoclonal phage ELISA, individual colonies of the transformed TG1 cells, produced after biopanning, were cultured in 1 mL of a 2xYT medium to an OD_600_ of 0.6, infected with a helper phage, and incubated at 30°C overnight. In parallel, the TcdB CROPs domain was immobilized onto the wells of an immunoassay plate (100 ng in 100 µL of a carbonate-bicarbonate buffer (CBB), pH 9.6, per well at 4°C for 16 h). The following day, the cells were pelleted by centrifugation and the supernatant containing recombinant phage particles was used for the analysis. The supernatant (50 μL) was added to each well of the plate and incubated at 37°C for 1 h. The wells were then washed with 300 μL of PBST five times, and a horseradish peroxidase-conjugated anti-M13 phage coat protein antibody (Sino Biological, China) was added.

To study the ability of single-domain antibodies to bind the CROPs domain, the CROPs domain was immobilized onto the wells of an immunoassay plate (100 ng in 100 μL of CBB per well at 4°C for 16 h). Then, serial three-fold dilutions of the antibodies in a blocking buffer, ranging in concentrations from 667 nM to 11.3 pM, were prepared, and 100 μL of each dilution was added to the wells of the plate. After washing the plate with PBST (300 μL per well, 4 times), a horseradish peroxidase-conjugated antiMyc-tag antibody (Abcam, UK) was added into the wells. Peroxidase activity was detected using a tetramethylbenzidine (TMB) solution, 100 μL per well. After incubation for 15 min, the reaction was stopped by adding 50 µL of 4 M sulfuric acid, and the optical density at 450 nm was measured.

**Production of single-domain antibody dimers**

The nucleotide sequences of dimeric forms of the single-domain antibodies were produced by two rounds of PCR according to the method described previously [16]. In the first round, the monomer sequences were amplified using Q5 High-Fidelity DNA Polymerase (NEB, UK). In the second round, the monomer sequences were fused to form dimers connected by a glycine-serine linker (Gly4 Ser)4 . The resulting PCR products were cloned into the pHEN vector. The antibody dimer sequences were verified by sequencing.

**Single-domain antibody expression and purification**

To express single-domain antibodies and their dimeric forms, Rosetta DE3 E. coli cells (NEB, UK) were transformed with phagemids carrying the antibody sequences. The transformed cells were cultured in a 2xYT medium containing ampicillin (100 μg/mL) at 37°C to an OD_600_ of 0.6, then IPTG was added to a concentration of 0.1 mM and the cells were incubated at 30°C overnight. The following day, the cells were pelleted by centrifugation and lysed using the BugBuster Protein Extraction Reagent (Novagen, USA). The recombinant antibodies were purified by metal affinity chromatography on an AKTA start instrument (Cytiva, USA) using a HisTrap column (Cytiva). Protein production levels and purity were analyzed using polyacrylamide gel electrophoresis.

**Expression and purification of recombinant C. difficile toxin B and its CROPs domain**

The sequences of TcdB and its CROPs domain were amplified using genomic DNA from the C. difficile strain VPI10463 (ATCC 43255) and cloned into the pHis1522 vector (MoBiTec GmbH, Germany).

To produce the recombinant TcdB and CROPs domain, the resulting constructs were transformed into Bacillus megaterium strain WH320 cells. The overnight culture was transferred to a 2xYT medium supplemented with tetracycline (10 μg/mL) and grown to an OD_600_ of 0.3. Then, xylose was added to the cells to a concentration of 0.5%, and the cells were incubated at 22°C overnight.

The cells were lysed by ultrasound, and the clarified lysate was purified by metal affinity chromatography using an AKTA start instrument and a HisTrap column. Protein yield and purity were analyzed by polyacrylamide gel electrophoresis.

**DNA isolation and Sanger sequencing**

Phagemid DNA was isolated from bacterial cells using a Plasmid miniprep 2.0 kit (Evrogen, Russia). Sequences encoding single-domain antibodies were sequenced using the Lac-prom (5’-CTTTATGCTTCCGGCTCGTATG-3’) and pIII-R (5’-CTTTCCAGACGTTAGTAAATG-3’) primers and a BigDyeTerminator 3.1 sequencing kit (Thermo Fisher Scientific, USA) on an Applied Biosystems 3500 genetic analyzer (Thermo Fisher Scientific, USA). Electrophoretic separation of DNA was performed in 50 cm capillaries with the POP7 polymer.

**In vitro TcdB neutralization assay**

The ability of the antibodies to neutralize TcdB was assessed in vitro using Vero E6 cells. Cells were seeded in 96-well plates (2 × 10^4^ cells/well) in 100 µL of DMEM supplemented with a 10% fetal bovine serum (FBS) and incubated at 37°C and 5% CO_2_ for 24 h. Threefold serial dilutions of the antibodies (from 200 nM to 0.1 pM, in 15 µL of DMEM medium with 10% FBS) were mixed with TcdB (10 ng/mL, in 15 µL of the DMEM medium with 10% FBS) and incubated at 37°C for 1 h. Then, the medium in the wells was replaced with a fresh medium (80 µL), 20 µL of a TcdB-antibody mixture was added, and the cells were incubated for 72 h. Intact Vero E6 cells and cells treated with TcdB without antibodies were used as controls. Cell viability was evaluated based on the cytopathic effect (phase-contrast microscopy) and ATP levels measured using a CellTiter-Glo kit (Promega, USA).

**Animal studies**

Female C57BL/6 mice (5- to 6-week-old) with a SPF status were used in the study. The study was approved by the Biomedical Ethics Committee of the Gamaleya National Research Center of Epidemiology and Microbiology of the Russian Ministry of Health in accordance with Protocol No. 68 of November 30, 2023. The experiments were conducted in compliance with ethical principles for working with laboratory animals, as stipulated by the European Convention for the Protection of Vertebrate Animals Used for Experimental and Other Scientific Purposes.

**Evaluation of the protective activity of the antibodies to TcdB**

The protective activity of the dimeric TB5A7 and TB4A8 antibodies was evaluated in a lethal TcdB challenge model using C56BL/6 mice. The animals were injected intraperitoneally with a pre-incubated (1 h, 37°C) mixture of a lethal TcdB dose (60 ng/mouse) and the dimeric TB5A7 and TB4A8 antibodies (500 μg/mouse). Control groups of animals were injected with 0.1% PBST (control group 1) or TcdB without antibodies (control group 2). The animals were observed for 120 h. Animals were excluded from the experiment upon reaching humane endpoints in accordance with bioethical standards.

**Wide-field fluorescence and confocal microscopy**

Cover slips sprayed with a monolayer of Vero E6 cells were fixed in 10% HistoSafe®-buffered formalin (BioVitrum, Russia) for 20 min. The actin cytoskeleton was stained according to the manufacturer’s protocol using Phalloidin AlexaFluor 488 (Invitrogen, USA); nuclei were stained with DAPI (AppliChem, Germany); embedding into the mounting medium was performed using 50% glycerol. Images were acquired using a ZEISS Celldiscoverer 7 fluorescence microscope equipped with an LSM 910 confocal module (Germany). The Alexa Fluor fluorochrome was excited by a laser at a wavelength of 493 nm (pinhole, 1 AU); DAPI was excited at a wavelength of 353 nm (pinhole, 1 AU); pixel size was 0.141 × 0.141 μm; a PlanApochromat 20 × 0.7 objective was used. The actin cytoskeleton was evaluated using four 150 × 150 μm fields of view for each experimental group. The actin cytoskeleton area was quantified using the ZEN 3.2 software (Germany) by measuring the total area of an Alexa Fluor 488 signal (μm^2^ ) per field of view; the signal was automatically segmented with thresholding using the Otsu method. The resulting value was normalized to the cell count (segmentation of nuclei and measurement of their number per field of view were also performed automatically using the Otsu threshold). Statistical analysis was performed in GraphPad Prism.

## RESULTS

**Fig. 1:**
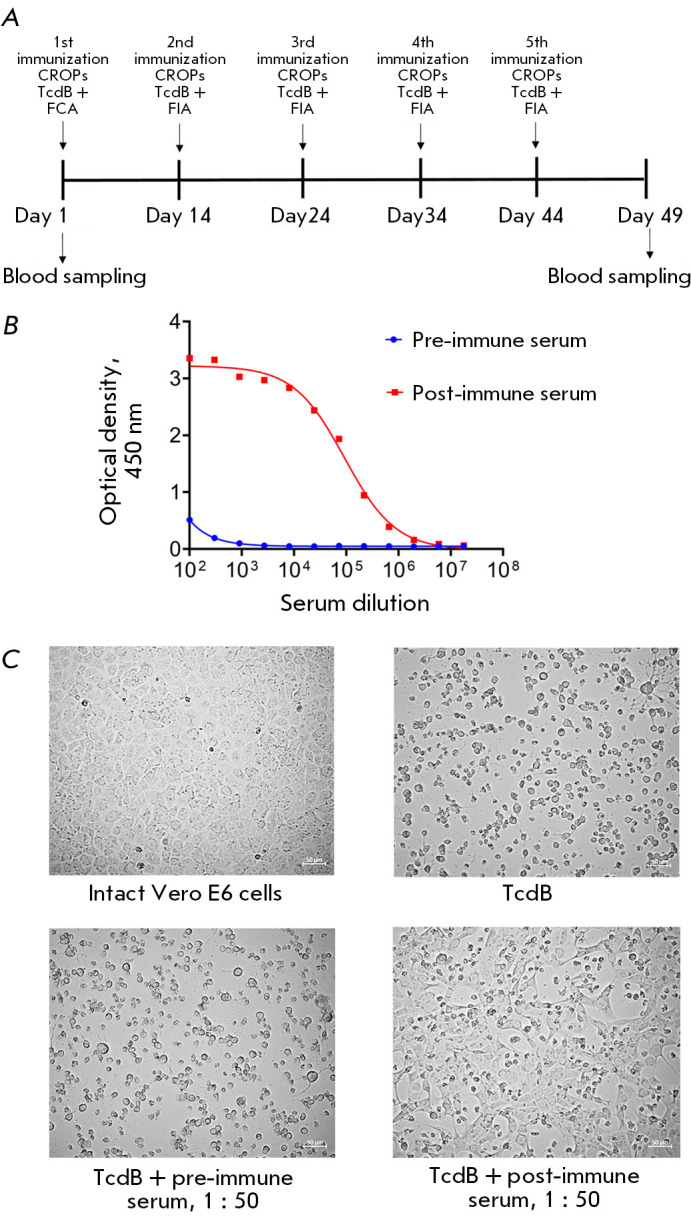
Alpaca immunization. (A) Alpaca immunization schedule. Immunization involved five consecutive injections according to the schedule. The recombinant TcdB CROPs domain of C. difficile strain VPI 10463 mixed with Freund’s complete adjuvant (FCA) or Freund’s incomplete adjuvant (FIA) was used as an immunogen. (B) Evaluation of the TcdB-specific antibody titer in the alpaca serum by ELISA. The CROPs domain was immobilized on immunoplates. Then, serial three-fold dilutions of pre-immune and post-immune serum samples were added into the wells. TcdB-bound antibodies were detected using HRP-conjugated anti-Llama IgG. (C) Analysis of the neutralizing activity of the alpaca serum. Images of intact Vero E6 cells, cells treated with TcdB (0.5 ng/mL), and cells treated with TcdB (0.5 ng/mL) pre-mixed with the pre-immune and post-immune serum samples (1 : 50 dilution) are shown

To generate neutralizing single-domain antibodies to C. difficile TcdB, we immunized an alpaca with the recombinant TcdB CROPs domain as an immunogen (Fig. 1A). The CROPs domain was produced in Bacillus megaterium. After immunization that involved five consecutive injections at 10- to 14-day intervals, a blood sample was collected. Analysis of the humoral immune response following immunization revealed a high serum antibody titer to toxin B, amounting to 1 : 1,970,000 (Fig. 1B). In vitro TcdB neutralization assay showed that the alpaca post-immune serum possessed pronounced neutralizing activity (Fig. 1C). Addition of the post-immune serum (dilution 1 : 50) to TcdB-treated cells resulted in 92% cell viability, whereas the alpaca pre-immune serum (dilution 1 : 50) exhibited no neutralizing activity.

**Fig. 2:**
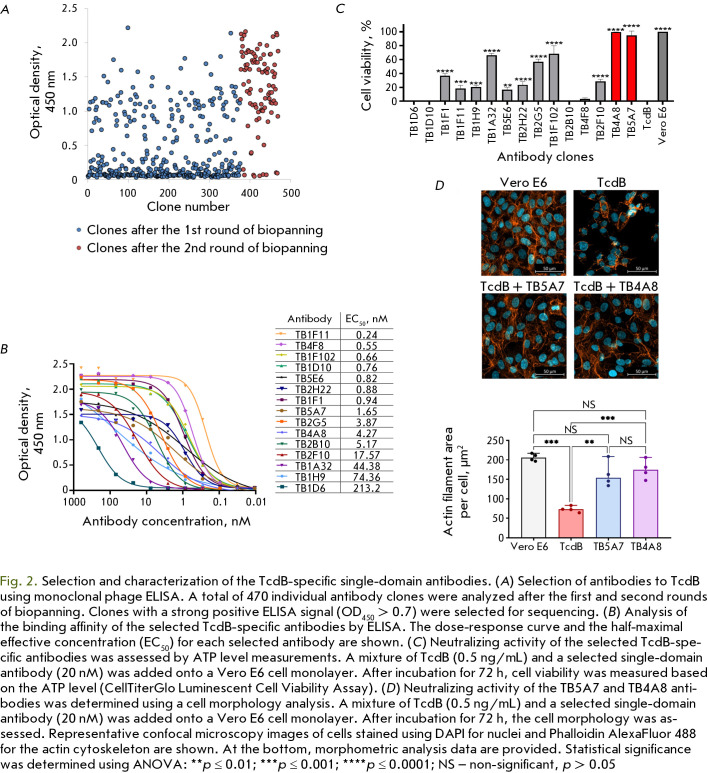
Selection and characterization of the TcdB-specific single-domain antibodies. (A) Selection of antibodies to TcdB using monoclonal phage ELISA. A total of 470 individual antibody clones were analyzed after the first and second rounds of biopanning. Clones with a strong positive ELISA signal (OD_450_ > 0.7) were selected for sequencing. (B) Analysis of the binding affinity of the selected TcdB-specific antibodies by ELISA. The dose-response curve and the half-maximal effective concentration (EC_50_) for each selected antibody are shown. (C) Neutralizing activity of the selected TcdB-specific antibodies was assessed by ATP level measurements. A mixture of TcdB (0.5 ng/mL) and a selected single-domain antibody (20 nM) was added onto a Vero E6 cell monolayer. After incubation for 72 h, cell viability was measured based on the ATP level (CellTiterGlo Luminescent Cell Viability Assay). (D) Neutralizing activity of the TB5A7 and TB4A8 antibodies was determined using a cell morphology analysis. A mixture of TcdB (0.5 ng/mL) and a selected single-domain antibody (20 nM) was added onto a Vero E6 cell monolayer. After incubation for 72 h, the cell morphology was assessed. Representative confocal microscopy images of cells stained using DAPI for nuclei and Phalloidin AlexaFluor 488 for the actin cytoskeleton are shown. At the bottom, morphometric analysis data are provided. Statistical significance was determined using ANOVA: **p ≤ 0.01; ***p ≤ 0.001; ****p ≤ 0.0001; NS – non-significant, p > 0.05

Next, peripheral blood mononuclear cells were isolated from the whole blood (50 ml) of the immunized alpaca. Sequences of the VHH domains of heavychain antibodies were cloned into a phagemid vector, and recombinant phagemids were transformed into E. coli TG1 cells. A VHH (single-domain antibody) library of 1.9 × 10^5^ individual clones was obtained. Then, recombinant bacteriophages exposing single-domain antibodies from the library were generated and antibodies to TcdB were selected using biopanning. A total of two rounds of biopanning were conducted, each followed by screening for TcdB-binding individual antibody clones using monoclonal phage ELISA (Fig. 2A). Clones showing strong positive ELISA signals (OD_450_ > 1.0) were selected for sequencing. A total of 470 individual clones were analyzed, and 21 unique clones of TcdB-binding antibodies were identified. These antibodies were expressed in E. coli Rosetta DE3 cells. The binding activity of the purified antibodies was analyzed by ELISA (Fig. 2B). Fifteen clones were found to bind to the CROPs domain, with half-maximal effective concentration (EC_50_) values ranging from 0.2 to 213.2 nM. Of these, 10 clones were characterized by high affinity, with EC_50_ values of less than 5 nM.

The neutralizing activity of the obtained singledomain antibodies against TcdB was evaluated using the Vero E6 cell line. Toxin B (0.5 ng/mL) was mixed with the antibodies (20 nM) and added onto a cell monolayer. Cell viability and cytoskeletal organization were assessed 72 h later. Two clones, TB4A8 and TB5A7, exhibited potent activity, with cell viabilities of 99.7 and 94.8%, respectively (Fig. 2C). Cell morphology analysis revealed that TcdB caused a significant reduction in the area of the cells, cell rounding, and loss of intercellular contacts (Fig. 2D). The addition of the TB4A8 and TB5A7 antibodies protected the cells from pathological changes. The morphometric analysis showed that the antibodies prevented TcdB-induced reduction in the actin filament content (Fig. 2D).

The next stage of the study involved the generation of dimeric forms of the two selected antibody clones, TB4A8 and TB5A7. The sequences of homodimers were obtained using molecular cloning methods. Expression and purification of dimeric forms was achieved using the same procedures previously described for monomers.

**Fig. 3:**
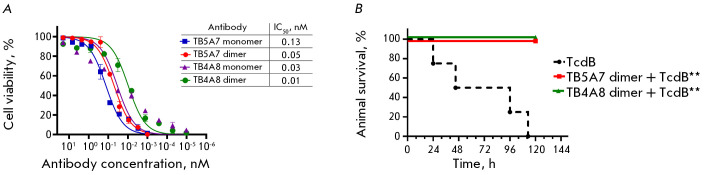
Neutralizing and protective activities of dimeric TB4A8 and TB5A7. (A) Comparison of the neutralizing activity of monomeric and dimeric forms of TB5A7 and TB4A8. TcdB (0.5 ng/mL) was mixed with serial dilutions of the antibodies and added onto a Vero E6 cell monolayer. After incubation for 72 h, cell viability was determined using the CellTiterGlo assay. The dose-response curve and the half-maximal inhibitory concentration (IC_50_) are presented. (B) Protective activity of dimeric forms of TB5A7 and TB4A8 after a lethal TcdB challenge. C57BL/6 mice were challenged with TcdB (60 ng per mouse) pre-incubated (37°C for 1 h) with the TB5A7 or TB4A8 dimer (500 μg per mouse). Kaplan–Meier survival curves are presented. Statistical significance was determined using the Mantel–Cox log-rank test (**p ≤ 0.01)

Comparative analysis of the neutralizing activity of the monomeric and dimeric forms of the antibodies was performed in the Vero E6 cell culture. Serial dilutions of the antibodies were mixed with TcdB and added onto a monolayer of the cells for 72 h. The dependence of cell viability on antibody concentration is shown in Figure 3A. Antibody dimers were found to exhibit higher neutralizing activity compared with that of monomers. The half-maximal inhibitory concentration (IC_50_) was 0.13 and 0.05 nM for the TB5A7 antibody monomer and dimer, respectively. For the TB4A8 antibody, the IC_50_ was 0.03 nM for the monomer and 0.01 nM for the dimer

In the final step, the protective efficacy of the selected antibodies was evaluated in a model of lethal TcdB challenge of mice. The dimeric TB5A7 and TB4A8 antibodies at a dose of 500 μg per mouse were mixed with a lethal dose of TcdB (60 ng/mouse) and administered to C56Bl/6 mice (Fig. 3B). It was discovered that both antibodies protected 100% of mice from a lethal challenge, whereas all TcdB-treated animals in the control group had died. These differences were statistically significant, with a p value of 0.0067

Overall, we isolated two single-domain antibodies, TB4A8 and TB5A7, possessing neutralizing activity against C. difficile toxin B. Dimeric forms of these antibodies provide 100% protection to mice from a lethal TcdB challenge.

## DISCUSSION

C. difficile is the most common cause of nosocomial antibiotic-associated diarrhea [17]. The incidence of CDI worldwide ranges from 1.1 to 631.8 cases per 100,000 population annually [18]. The recommended treatment for CDI includes the use of three antibacterial drugs: vancomycin, metronidazole, and fidaxomicin. However, the spread of resistant strains, high recurrence rates, and significant mortality rate have fueled the development of novel effective therapeutic strategies. For example, fecal transplantation and probiotic agents are used as alternatives [19, 20]. One of the promising approaches is the development of agents that neutralize the effect of the key virulence factors of the bacteria, toxins A and B. These agents include monoclonal antibodies that bind to and neutralize bacterial toxins. Currently, one agent based on monoclonal anti-TcdB antibodies, bezlotoxumab, has been approved. Its use in combination with antibiotics reduces the recurrence rate of CDI [21].

This study was devoted to the isolation of monoclonal single-domain antibodies to C. difficile toxin B. Single-domain antibodies are the variable domain of heavy-chain antibodies found in certain animal species, including all members of the Camelidae family [22]. The development of therapeutics based on single-domain antibodies has several advantages associated with the unique properties of these antibodies, in particular their high thermal stability, ability to bind to epitopes non-accessible to conventional antibodies, and simplicity of generation of multimeric molecules [22, 23]. To isolate single-domain antibodies to TcdB, we performed alpaca immunization with a recombinant CROPs domain. This fragment was chosen as an immunogen, because it exhibits no toxic effect and is also involved in TcdB attachment to the target cell via interactions with surface glycans [6]. Analysis of the neutralizing activity of the alpaca post-immune serum ACell viability, % Antibody concentration, nM Animal survival, % Time, h TcdB TB5A7 dimer + TcdB** TB4A8 dimer + TcdB** Antibody IC_50_, nM TB5A7 monomer 0.13 TB5A7 dimer 0.05 TB4A8 monomer 0.03 TB4A8 dimer 0.01 100 80 60 40 20 0 100 80 60 40 20 0 10 0 24 48 72 96 120 144 1 100 10-1 10-2 10-310-4 10-5 10-6 B Fig. 3. Neutralizing and protective activities of dimeric TB4A8 and TB5A7. (A) Comparison of the neutralizing activity of monomeric and dimeric forms of TB5A7 and TB4A8. TcdB (0.5 ng/mL) was mixed with serial dilutions of the antibodies and added onto a Vero E6 cell monolayer. After incubation for 72 h, cell viability was determined using the CellTiterGlo assay. The dose-response curve and the half-maximal inhibitory concentration (IC_50_) are presented. (B) Protective activity of dimeric forms of TB5A7 and TB4A8 after a lethal TcdB challenge. C57BL/6 mice were challenged with TcdB (60 ng per mouse) pre-incubated (37°C for 1 h) with the TB5A7 or TB4A8 dimer (500 μg per mouse). Kaplan–Meier survival curves are presented. Statistical significance was determined using the Mantel–Cox log-rank test (**p ≤ 0.01) revealed that the serum, at a 1 : 50 dilution, neutralized TcdB, whereas the pre-immune serum lacked protective capacity.

In the next stage of the study, a panel of single-domain antibodies to TcdB was obtained. Two of these, TB4A8 and TB5A7, exhibited the most pronounced neutralizing activity against the toxin and were selected for further research. To enhance their neutralizing activity, we produced bivalent forms of the antibodies that comprised two monomers connected by a flexible glycine-serine linker. Dimerization of singledomain antibodies is used to increase the affinity of antibody interaction, which improves their functional activity. Furthermore, a larger size of the dimer molecules compared with that of monomers increases the half-life of the antibodies in in vivo experiments. Analysis of the neutralizing activity of the TB4A8 and TB5A7 antibody dimers revealed their increased activity compared with that of monomers. The neutralizing activity of the TB5A7 and TB4A8 dimers increased 2.5-fold and 3-fold, respectively, compared with that of the corresponding monomeric forms. The increased neutralizing activity of multimerized antibodies to C. difficile toxins has also been noted by other authors. For example, a study by Zhiyong Yang et al. demonstrated a significant increase in the neutralizing activity of a multivalent antibody, ABA, to TcdA [24].

Next, we investigated the protective activity of the produced bivalent antibody forms in an in vivo model. The TB4A8 and TB5A7 antibody dimers were shown to fully protect mice from a lethal toxin B challenge. The survival rate of the animals in groups receiving the antibodies (500 μg/mouse) was 100%, whereas all the animals in the control group died. These findings demonstrated the potential of the isolated monoclonal antibodies to protect against the severe symptoms of a C. difficile infection associated with the action of TcdB. [10]. Thus, the TB4A8 and TB5A7 antibodies are promising candidates for clinical application.

## CONCLUSION

The toxins A and B are the main virulence factors of C. difficile. Thus, they are of interest as therapeutic targets in a C. difficile infection. In this study, we isolated monoclonal single-domain antibodies that display neutralizing activity against TcdB and protect animals from a lethal dose of the toxin. The study is an important step in the development of novel therapeutics for the treatment of CDI, which may reduce morbidity and mortality from this infection.

## Abbreviations

VHH: variable domain of heavy-chain-only antibody
CDI: Clostridioides difficile infection
TcdA: C. difficile toxin A
TcdB: C. difficile toxin B
CDT: C. difficile transferase (binary toxin)
GTD: glucosyltransferase domain
APD: autoprotease domain
DD: delivery domain (translocation domain)
CROPs: combined repetitive oligopeptides domain
PBS: phosphate-buffered saline
IPTG: isopropyl β-D-1-thiogalactopyranoside
